# A Tool for Assessing a Community’s Capacity for Substance Abuse Care

**DOI:** 10.5888/pcd13.160190

**Published:** 2016-09-22

**Authors:** Brandn Green, Rob Lyerla, Donna F. Stroup, Alejandro Azofeifa, Patrick M. High

**Affiliations:** Author Affiliations: Rob Lyerla, Alejandro Azofeifa, Patrick M. High, Center for Behavioral Health Statistics and Quality, Substance Abuse and Mental Health Services Administration, Rockville, Maryland; Donna F. Stroup, Data for Solutions, Inc, Decatur, Georgia.

## Abstract

Evidence-based programs for prevention and intervention in substance abuse are increasing. Community needs assessments and health rankings provide descriptions of local behavioral health needs but do not provide public health practitioners and policy makers with guidelines on the number of programs, health care practitioners, or interventions needed in the local substance abuse care system. This article presents a new framework for measuring and assessing the substance abuse care system in a community. The assessment can inform resource allocation across the continuum of care to more equitably and efficiently distribute interventions and care. We conducted 2 literature reviews and synthesized our findings to create a community assessment methodology and needs calculator, CAST (calculating for an adequate system tool). We reviewed 212 articles to produce an inventory of community and social correlates of behavioral health, components of a substance abuse care system, and numerical values for guidelines for estimating community needs. CAST produces community-specific assessments of the capacity of the components of a community substance abuse care system. CAST generates recommendations by the application of social and community determinants of health as risk coefficients to each estimate of component need. CAST can assist public health practitioners in evaluation and improvement of the capacity of community-based, substance abuse care systems. By using recommendations for component needs across the continuum of care, community leaders can use CAST to prioritize resource allocation more effectively and efficiently.

## Introduction

Behavioral health problems are increasing the health care burden in the United States. The age-adjusted death rates from opioid overdose in the United States rose from 1.5 in 2000 to 5.4 in 2010, and then fell to 5.1 in 2013. The age-adjusted death rate for heroin-related drug poisoning nearly tripled from 2010 to 2013, from 1.0 to 2.7 per 100,000 (1). The overall age-adjusted death rate from chronic liver disease and cirrhosis in the United States rose from 1999 through 2013, from 9.6 to 10.2 per 100,000. Age-specific death rates, however, have been increasing among adults aged 45 to 64, from 17.4 to 20.1 per 100,000 for those aged 45 to 54 and from 23.7 to 30.4 per 100,000 for those aged 55 to 64. Patterns in death rates were similar for the subset of people with alcohol-related liver disease (2). The overall 2013 death rate from all alcohol-related causes was 9.2 per 100,000, with the highest rate by age of 25.3 per 100,000 among those aged 55 to 64. Of these deaths, 74% were men, and 86% were non-Hispanic whites (3). These trends in behavioral health problems are in contrast to gains in life expectancy and reduction in mortality from many other causes.

Behavioral health problems have an economic impact in the United States. For example, according to Sacks et al (4), “excessive [alcohol] drinking cost the US $249 billion in 2010 . . . a significant increase from $223.5 billion . . . in 2006. Most of these costs were due to reduced workplace productivity, crime, and the cost of treating people for health problems caused by excessive drinking.” Opioid abusers generate, on average, annual direct health care costs 8.7 times higher than costs generated by nonabusers (5).

In 2006, an estimated $57.8 billion was spent in the United States on care for mental illness, which is similar to expenditures on cancer care (6). Worldwide, the economic impact of mental illness for the period from 2010 through 2030, calculated 3 different ways, is estimated to be similar to that of cardiovascular disease (7).

Despite state and community planning efforts, behavioral health care systems lack sufficient capacity for addressing the needs of the population they serve. These systems were developed in the midst of funding shortages, shifting health care priorities, and decentralized planning efforts and by multiple organizational stakeholders (8). As a result, community behavioral health care systems have gaps in comprehensive care and redundancy of resource allocation (9). These inefficiencies can be addressed to improve the composition of local behavioral health care systems. Assessing the local system requires both a framework for defining an adequate care system and a method for estimating demand for each component of the system to address the behavioral health care needs of the community population (10).

To our knowledge, a comprehensive framework for a community-wide substance abuse care system has not been articulated. Nor have social and community indicators been used to mathematically produce estimations of units of need for the baseline components of a behavioral health care system. In this article we describe development of CAST (calculating for an adequate system tool), which provides both the framework for a localized behavioral health care system and an equation for estimating needs for each component of the framework. Rather than focusing on characteristics of the people at risk, our methodology provides a comprehensive assessment of the community’s health care infrastructure for substance abuse. The theory and methodological approach used to develop CAST could be applied to structure other services-based, community-wide assessments or evaluations at the systems level.

## Methods

CAST is based on an expansion of the widely accepted Substance Abuse and Mental Health Services (SAMHSA) continuum of care (11). We added a category, referral, to the continuum-of-care model to more fully depict a local system of care. The 5 categories along the continuum that we used for CAST were promotion, prevention, referral, treatment, and recovery.

To produce the framework for an adequate care system, we systematically searched the literature. Because this work was exploratory, a systematic review was not possible. We reviewed 75 articles about prevention, promotion, referral, treatment and recovery programs, interventions, and medical professionals as these related to behavioral health. This review identified the components necessary for an adequate system of comprehensive substance abuse and for each component of the system, dosage rates, use rates, and treatment group sizes. When no guidelines for benchmarks of dosage rates, use rates, or treatment group sizes were available from the literature, we estimated benchmarks by using the median rate or size as observed in national surveys of service provision and use.

We used Web of Science and PubMed to systematically search the literature to identify relevant social and community indicators of substance abuse according to 3 criteria agreed upon by the authors: 1) research findings displayed a consistent relationship between the indicator and a high likelihood to engage in substance abuse, 2) research findings displayed a consistent relationship between the indicator and a high likelihood to engage in substance abuse treatment, and 3) data about social indicators were available at the county level. Each article was evaluated by a single reviewer. We evaluated and catalogued 143 articles, including 15 meta-analyses of research on social and community correlates of substance use and abuse. Sixty-three possible indicators were identified through the review process; of these, 18 met the review criteria for inclusion.

CAST can calculate community-specific recommendations on the need for each of the promotion, prevention, referral, treatment, and recovery components by using the prevalence of social and community correlates of substance abuse to modify estimates of the population’s needs. This process is based on accepted models to inform decision making that use mathematical assumptions about the impact of factors related to a desired outcome (12) (eg, the effect of screening programs for sexually transmitted infection on subsequent transmission of infection, the usefulness of condoms in pregnancy prevention) (13,14). Each calculation of a component need includes values for the total target population, individual treatment exposure, individual dosage frequency (in a year), and number of treatment group participants. The following equation estimates the CAST community need for each component along the continuum of care:

Community Need = [(*X*_1_ × *Y*_1_)/(*Z*_1_)] × [1 + *R*] × *U*

Where:

*X*_1_ = Total target population
*Y*_1_ = Individual dosage frequency (in a year)
*Z*_1_ = Number of treatment group participants
*R* = Prevalence ratio of social indicators of substance abuse + Prevalence ratio of community indicators of substance abuse
*U* = Usage rate or percentage of target population expected to use the component

The social and community risk coefficient adjustment (1 + *R*) in the equation produces recommendations for total component needs that reflect the characteristics of the people in the service area providing substance abuse care.

## Using CAST to Assess Community Needs

We identified 32 components of a behavioral health care system, organized them by the modified 5 continuum-of-care categories, and used them to create CAST (Table 1). The indicators of substance abuse used to produce the community risk score were grouped by type: demographics (descriptive statistics about population and community characteristics), social indicators (aggregated ordinal variables used to describe the characteristics of people living in a geographically defined region), and community indicators (categorical variables that affect social context regardless of demographic or social characteristics) (Box).

**Table 1:** CAST Framework for Assessing a Community’s Capacity for Substance Abuse Care

Continuum-of-Care Category Component	Type of Intervention
**Promotion**	
Social marketing campaign	Campaign
Media advocacy events	Event
Community coalitions	Coalition
**Prevention**	
School-based programs	Single program event
Community-based programs	Single programs event
Faith-based programs	Short-term program
Workplace programs	Short-term program
Housing vouchers	Voucher
Needle exchanges	Needle exchange location
Prescription drug disposal locations	Drop off location
**Referral**	
Adult drug courts	Drug court
Youth drug courts	Drug court
Social workers	Social worker
Crisis-intervention–trained police	Police officer
Employee assistance programs	Program
Primary care medical providers with specialty training in substance abuse	Health care professional
**Treatment**	
Inpatient detoxification	Admissions
Inpatient 24-h/intensive day treatment	Program
Inpatient short-term (30 days or fewer)	Program
Inpatient long-term (more than 30 days)	Program
Outpatient detoxification	Admissions
Counselors, psychiatrists, or psychotherapists	Health care professional
Office-based opiate substitution	Program
**Recovery**	
Religious or spiritual advisors	Religious community professional
12-step groups	Meeting
Peer support groups	Group
Transportation	Round trip ride
Employment support	Social service professional
Educational support	Class
Parenting education	Class
Housing assistance	Social service professional
Insurance assistance	Certified application counselor

Abbreviation: CAST, calculating for an adequate system tool.

**Box. Social and Community Indicators of Substance Abuse Used in CAST (Calculating for an Adequate System Tool)**

**Demographics**

Age: percentage aged 10–19 y and percentage aged 20–65 y

Sex: percentage male or female

Total population

**Social Indicators**

Voter turnout <35% (15)

High school dropout rate >12% (16)

Homeless population >2% (17)

Incarceration rate >1.5 per 100 people (18)

Veteran population >2,000 in the county (19)

Previously in foster care rate >5 per 100 people (20)

More than 12% of households with income <$35,000 (21)

Median household income >$53,000 (15)

More than 30% have a college degree (22)

Divorced, widowed, separated rate >3.5 per 1,000 in past year (23)

Percentage uninsured >20% (24)

**Community Indicators**

County designated as a high-incidence drug trafficking area (25)

Alcohol outlet density >0.4 liquor stores per 10,000 people (26)

Collapse of a major employer (27)

Presence of a university (28)

Presence of a military base (29)

Violent crime rate >300 per 100,000 people (15)

Access to exercise >50% (30)

Thresholds for each indicator were identified that corresponded to research findings or, when no thresholds for community-wide effect existed, national medians were calculated and used to indicate when social or community rates or proportions would be expected to increase risk for substance abuse in the community.

### Guidelines for implementing CAST

Local estimates of substance use and substance abuse are difficult to calculate because communities may vary in the precision and extent of their data infrastructure. For CAST, “community” is defined as the geographic area selected by the user of the tool. If a community does not have precise substance use and abuse data, the subregional estimates from the National Survey on Drug Use and Health (NSDUH) are used as proximate measures of local prevalence (31). The subregional estimates of NSDUH are a more precise reflection of a community, even accepting local variation, than national estimates at the local level. The percentage of expected substance users is multiplied by the total population of the community aged 12 to 65 to produce a total estimate of users in need of care at some point in the local system.

The total population estimate does not reflect the demographic, social, and community correlates of substance abuse that constitutes the total population in need of services. To address this limitation, the community undertaking the assessment is given a score based on the prevalence of selected social and community indicators. This score is defined as the social and community determinants risk. This risk score is applied to the component need estimates for each component in the community. Applying this coefficient adjusts the component need estimates to reflect the substance use tendencies of the populations living in the community.

CAST initially produces a maximum community need for 100% coverage of everyone who may interact with a given component of the care system. This preliminary total is an overestimation of community need, because use rates for individual components vary greatly. A national study demonstrated that for any given component of a substance abuse care system, only a fraction of the total available user base will participate (32). Participation rates were determined by identifying values derived from research evaluating participation rates in system components or categories of care, or by using national estimates of participation from the National Survey of Substance Abuse Treatment Services (N-SSATS). The maximum community need can be multiplied by the use rate to produce the recommended number of component units (Table 1) needed to meet the needs of the component-using population. The fundamental assumption of this approach is that an adequate and complete substance abuse care system is achieved when a community can provide services to the population accessing services, not the entire population needing services.

Currently, CAST is implemented in Microsoft Excel. The Excel spreadsheet (Appendix) organizes and applies the elements of CAST presented in this article and is available as a download for use. Additional information, including all supporting citations, is available upon request to the corresponding author.

### Example

Currently, 3 pilot studies of CAST application are underway in different types of communities in the United States. These pilot studies are being conducted through collaborations with local public health and behavioral health departments in a major city, a public health region consisting of 10 counties, and a single rural county. Pilot studies will evaluate the accuracy of CAST’s estimates and recommendations of component need and the comprehensiveness and accuracy of the framework for a community-wide substance abuse care system. To demonstrate how CAST functions, we compared 2 hypothetical communities to demonstrate how the algorithm produces need estimates for each component of the substance abuse care system (Table 2). For ease of comparison, the total population, sex composition, and age compositions of these communities were held constant.

**Table 2:** Comparison of CAST Applied to 2 Hypothetical Communities of 50,000 by 32 Components of a Community Substance Abuse Care System and by Risk Score^a^

Continuum of Care Category Component	Adjusted Community Need^b^, No. of Units Needed		Type of Intervention
	Community A Population, Risk Score of 0.78^c^	Community B Population, Risk Score of 1.06^c^	
**Promotion**			
Social marketing campaign	20	27	Campaign
Media advocacy events	7	10	Event
Community coalitions	1	2	Coalition
**Prevention**			
School-based programs	54	74	Single program event
Community-based programs	48	66	Single program event
Faith-based programs	4	6	Short-term program
Workplace programs	1	1	Short-term program
Housing vouchers	27	36	Voucher
Needle exchanges	1	2	Needle exchange location
Prescription drug disposal locations	9	13	Drop off location
**Referral**			
Adult drug courts	7	9	Drug court
Youth drug courts	2	2	Drug court
Social workers	3	4	Social worker
Police trained in crisis intervention	0	0	Police officer
Employee assistance programs	1	1	Program
Primary care medical providers with specialty training in substance abuse	17	24	Health care professional
**Treatment**			
Inpatient detoxification	11	14	Admissions
Inpatient 24-hour intensive day treatment	35	48	Program
Inpatient short-term treatment (≤30 days)	13	17	Program
Inpatient long-term (>30 days)	21	29	Program
Outpatient detoxification	11	14	Bed
Counselors, psychiatrists, or psychotherapists	20	27	Health care professional
Office-based opiate substitution	1	1	Program
**Recovery**			
Religious or spiritual advisors	1	1	Individual
12-step groups	106	144	Meeting
Peer support groups	64	86	Group
Transportation	154	210	Round trip ride
Employment support	3	4	Social service professional
Educational support	32	43	Class
Parenting education	14	19	Class
Housing assistance	1	2	Social service professional
Insurance assistance	10	13	Certified application counselor

Abbreviation: CAST, Calculating an Adequate System Tool.

a Risk score is 1 + the prevalence ratio of social indicators of substance abuse + the prevalence ratio of community indicators of substance abuse.

b Adjusted community need is recommended number of component units needed to meet the needs of the component-using population.

c Population, 50,000.

## Discussion

The CAST methodology is both a framework for assessment of the substance abuse care system and a strategy for producing estimates of what is lacking and what is redundant for the 32 components of the 5 main component categories (promotion, prevention, referral, treatment, recovery) in a continuum of care. Comparing observed totals of system components with recommended components facilitates making evidence-based decisions at the community level. Community health systems are most effective when they integrate current expertise from the research community (33).

The strengths of CAST are that it describes the information required to assess needs and bases its calculation on best evidence available from the current scientific literature. State-of-the-science methods are built into the values used to calculate the community recommendations; the math required to accurately weight the social determinants reflects the current trends in health modeling and analytics. The methodology can be adapted as needs and characteristics of the problem of substance abuse change.

A limitation of CAST is the accuracy of the values used to estimate need for each component in the system. Evidence supporting decisions about benchmarks and program dosage rates is limited. Although median rates are useful for estimating benchmarks, they may be less precise than those produced through subsequent research (34). These limitations would be addressed by refining and adjusting the thresholds as understanding improves about the effect social and community indicators have on community-wide substance abuse behaviors. In subsequent iterations of CAST, the singular thresholds will be replaced with ranges to more accurately reflect the social determinants of behavioral health. Work is in progress to evaluate the sensitivity of these determinants to variation and modification via Monte Carlo simulations (35). Monte Carlo simulation is a problem-solving technique that relies on random sampling and statistical modeling to determine the probability of certain outcomes. It is most useful when experimentation is too time-consuming, costly, or impractical to perform. We will randomly vary the components of CAST and assess the stability of the results. In addition, confidence intervals will be calculated for each of the recommendations for component needs.

Developing tools to enable assessments of health systems is the next stage in community health needs assessments, because the tools allow for informed planning about services designed to meet the needs of community-specific populations. By using a mathematical relationship between the presence of social and community determinants of substance abuse, CAST provides a quantitative method for making recommendations responsive to the social conditions in which they are enacted.

CAST produces an assessment of a community substance abuse system by calculating need or excess for selected evidence-based components in the continuum of care. Community leaders can use CAST to inform decisions about financial, human, and infrastructure resource allocation to address substance abuse in their communities. By identifying redundancies and gaps, CAST provides an assessment framework and community-specific guidelines for component need. A more carefully distributed substance abuse care system will decrease substance abuse rates by linking services with community need more effectively and precisely.

CAST will be most effective when it is integrated into local processes of identifying the goals community members and leaders have for how they would like to see substance use managed. One community may have a preference for prevention among school children, while another might have a preference for recovery supports for former addicts. The public health practitioners who use CAST will be able to accept, reject, or adjust the recommendations presented by the tool in accordance with their community priorities. The estimated need totals are guidelines to enable more informed decision making, not dogmatic guarantees of community-wide success. This tool is intended to promote local collaboration, intentional resource allocation, and strategic planning.

## Appendix Social and Community Determinants Interface for CAST (Calculating for an Adequate System Tool).

To use the CAST interface for estimating the community risk score, users should fill in all cells on the Community Characteristics tab, starting with age and sex ratios for their community and the total population of the community. Users should then place a 1 in the “yes” or “no” cell for each indicator that reflects the characteristics of their community. The tool will calculate the risk score, which is applied to produce estimates for the 32 components of CAST. Under the Capacity Calculator tab, users will see the estimated community needs for each component. To fully use the CAST method, users should collect prevalence estimates for each component in their community and add these totals to the Observed Community Totals column. When all of the necessary information has been inserted by the user, the Estimated Need totals tell the user if their community has an excess or deficit for each component type.

This spreadsheet is available for download as a Microsoft Excel file.

An Adobe PDF version of this spreadsheet is available for download.

People with disabilities experiencing problems accessing the materials on this web page should contact Brandn Green, PhD, [Brandn.green@samhsa.hhs.gov].
