# Supplementary material for: A Tool for Assessing a Community’s Capacity for Substance Abuse Care
Source: Prev Chronic Dis. 2016 Sep 22;13:E133. doi: 10.5888/pcd13.160190 (PMC5034557; doi:10.5888/pcd13.160190)
Supplement: Supplementary file 2 [file 16_0190_Appendix_508.pdf]

Substance Abuse Service Capacity Calculator

Community Name

Color Codes

- Default values
- Calculated by the model
- Added by Community Member

Demographics	% Age 10-19	% age 20-65		Total Population
Female				
Male				

Social Indicators of Behavioral Health	Voter Turnout below 35%	High school drop out greater than 12%	Homeless Population above 2%	Incarceration rate above 1.5 per 100 people	Veteran population above 2750 in your county	Previously in foster care rate above 5 per 1000 people	More than 12% of households with income below $35,000	Median household income above $53,000	More than 30% with college degree	Divorced, Widowed, Separated rate above 3.5 per 1000 people in past year	Percent uninsured above 20%	*Total Score*
Yes												
No												0.000

Community Indicators of Behavioral Health	County designated as High Incidence Drug Trafficking Area	Alcohol Outlet Density over .4 liquor stores per 10,000 people	Collapse of a major employer	University	Miltary Base	Violent Crime Rate above 300 per 100000	Walkability Score below 50	*Total Score*
Yes								
No								0.000

Questions? Contact: Brandn Green - brandn.green@samhsa.hhs.gov

Intervention	Definition
Advertisements	intentional, informational campaigns that use advertising theories to alert community of a substance use problem and/or treatment program. Medium of the advertisement is not relevant for effectiveness, will likely influene the popluation seeing the campaign.
Advocacy Events	Media campaign with a specific, community change agenda related to behavioral health or management of substance use. Garnering local news attention to move forward the cause is a key consideration.
Coalitions	Any intentional collective of local organizational leaders, be they poliltical, non-profit, or business organizations, receiving and allocating grant funding to limit substance use, abuse, and/or dependence. To be the most effective, research has shown that a highly capable board of directors, a commitment to research & evidence-based practices, and the hiring of a dedicated staff person correlate with a positive impact on lower usage rates.
School.Prevention	1 hour long, drug/alcohol, usage and prevention educational programming taking place within a school setting.
Communinty.Prevention	Prevention programs taking place within non-profit or social services settings.
Faith.Prevention	Prevention programs taking place within a religious community
Workplace.Prevention	Programs for primary prevention of alcohol abuse in the workplace…which focus on changing individual behavior, as well as environmental interventions, to reduce risk factors by changing the work enviornment.
Mobile Outreach	Mobile phone based reminder services about groups and treatments for individuals undergoing treatment or who have participated in groups.
Housing Vouchers	Voucher programs to enable low-income residents higher degrees of neighborhood mobility.
Needle Exchange	A social service that allows injecting drug users to obtain hpodermic needles and associated paraphernalia at little or not cost
P.Drug disposal	Programs that inform the general public about safe storage and disposal of prescrpition drugs; collection of drugs by officials at permanent return programs or one-day events
Adult Drug Courts	Provision of substance abuse treatment in combination with collaborative case management and supervision. Adult Drug Court model used for estimation. Other types of court models exist and might also be relevant for the community.
Primary Care Doctors	Primary care doctors who have received or attended training in substance abuse recognition and are willing to engage in brief interventions

Social Workers	Social workers who have received education or training related to the identification of substance abuse and/or mental health
Youth Drug Courts	Provision of substance abuse treatment in combination with collaborative case management and supervision as an alternative to incarcertaion for juveniles
MH awareness police training	Police officers serving a community who have received additional education and training on how to recognize and respond to mental health needs.
Employer/EAP	Assessment, short-term counseling, and referral services to employees with substance abuse and other work-related problems
Detox - inpatient	24 hour per day medical acute care services in hospital or residentiall setting for safe withdrawl and transition to ongoing treatment. Count both hospital and residential based detox locations
24 intensive/Intensive Day	A nonresidentaial, psychiatric care program
Short term (30 days or fewer)	Less than 30 days of non-acute care in a setting with treatment services for alcohol and other drug abuse and dependency
Long term (more than 30 days)	More than 30 days of non-acute care in a setting with treatment services for alcohol and other drug abuse and dependency; this may include transitional living arrangements such as halfway houses
Detox - outpatient	A period of medical treatment during which a person is helped to overcome physical and psychological dependence on alcohol occuring in an outpatient setting
MH Professionals	A certified provider of psychiatric and mental health counseling, medical services, and care.
Opiate therapy	Medical providers of opiate substitution therapies such as methadone or buprenorphine
Rel.advisors	Religious professionals providing substance abuse therapy and counseling
12-step groups	Brief, structured, and manual-driven approaches to treatment
Peer support groups	A voluntary gathering of people with similar challenges, related to substance use/abuse, meeting weekly or monthly for an hour or two, to share experiences and coping strategies.
Transportation	Programs provided by treatment facilities or community center to aid recovering individual in accessing treatment.
Employment support	Programs explicitly aimed at assisting post-treament, recovering, community members gain access to employment.
Educational support	Adult education programs aimed at helping recovering community members to achieve educational goals, i.e., get an GED.
Parenting education	Classes designed to educate adults about the many issues children face from the effects of substance abuse.

Housing assistance	Programs aimed at finding housing for individuals in recovery. They may or may not include a specific treatment component.
Insurance access assistance	Insurance assistance available to individuals in recovery.

Intervention	Usage Rate
Advertisements	85%
Advocacy Events	30%
Coalitions	100%
School.Prevention	93%
Communinty.Prevention	12%
Faith.Prevention	9%
Workplace.Prevention	34%
Mobile Outreach	53%
Housing Vouchers	42%
Needle Exchange	60%
P.Drug disposal	60%
Adult Drug Courts	50%
Primary Care Doctors	10%
Social Workers	71%
Youth Drug Courts	50%
Police	25%
Employer/EAP	2%
Detox - inpatient	1%
24 intensive	2%
short term	2%
long term	6%
Detox - outpatient	1%
MH Professionals	74%
Opiate Therapy	25%
Rel.advisors	2%
12-step groups	30%
Peer support groups	9%
transportation	14%
employment support	5%
Educational support	14%
Parenting education	7%
Housing Assistance	7%
Insurance Access Assistance	90%

Intervention	Treatment Group Size
Advertisements	5000
Advocacy Events	5000
Coalitions	30,000
School.Prevention	300
Communinty.Prevention	100
Faith.Prevention	238
Workplace.Prevention	30
Mobile Outreach	1000
Housing Vouchers	25
Needle Exchange	2000
P.Drug disposal	1200
Adult Drug Courts	125
Primary Care Doctors	20
Social Workers	30
Youth Drug Courts	125
Police	5880
Employer/EAP	27
Detox - inpatient	5
24 intensive	3
short term	10
long term	15
Detox - outpatient	5
MH Professionals	27
Opiate Therapy	187
Rel.advisors	27
12-step groups	30
Peer support groups	15
transportation	15
employment support	15
Educational support	15
Parenting education	15
Housing Assistance	15
Insurance Access Assistance	50

Intervention	Individual Treatment Exposure	Value for Calculation
Advertisements	3 views per ad	3
Advocacy Events	5 substances. 3 events per year	3
Coalitions	N/A	
School.Prevention	3 events per year	3
Communinty.Prevention	1 program per year	1
Faith.Prevention	1 program per year	1
Workplace.Prevention	1 program per year	1
Mobile Outreach	One alert each week	52
Housing Vouchers	2 vouchers to provide assistance for housing per year	2
Needle Exchange	Once a month per year	12
P.Drug disposal	Once a year	1
Adult Drug Courts	1.4 per year	1.4
Primary Care Doctors	Once a year	1
Social Workers	Need to find ave. length of involvement	1
Youth Drug Courts	1.4 per year	1.4
Police	Could find something related to exposure/interaction with police	1.4
Employer/EAP	Once a year	1
Detox - inpatient	Once a year	1
24 intensive	Once a year	1
short term	Once a year	1
long term	Once a year	1
Detox - outpatient	Once a year	1
MH Professionals	Once a year	1
Opiate Therapy	One a year	1
Rel.advisors	Need to estimate.	1
12-step groups	Twice a year	2
Peer support groups	Twice a year	2
transportation	Once a week	52
employment support	Three a year	3
Educational support	Classroom program - 2 per year at 20 weeks per class	2
Parenting education	Classroom program - 10 classes per program	1
Housing Assistance	Three times a year	3
Insurance Access Assistance	Once a year	1

Intervention	Population	Value
Advertisements	Community	0
Advocacy Events	Community	0
Coalitions	Community	0
School.Prevention	Population % under 18	0
Communinty.Prevention	Community	0
Faith.Prevention	Community % that participate in faith communities	0
Workplace.Prevention	Work force with heavy alcohol use (8.8%, Ames 2011)	0
Mobile Outreach	Substance Users seeking treatment	0
Housing Vouchers	Low-income Users Seeking Treatment	0
Needle Exchange	IV Drug Users	0
P.Drug disposal	Community members with opioid prescriptions	0
Adult Drug Courts	Adults arrested for Drug use	0
Primary Care Doctors	Substance Dependent/Abuser	0
Social Workers	Substance Dependent/Abuser accessing social workers	0
Youth Drug Courts	Juveniles arrested for drug use	0
Police	% of substance using population that interact with police	0
Employer/EAP	Work force with heavy alcohol use	0
Detox - inpatient	Users seeking treatment	0
24 intensive	Users seeking treatment	0
short term	Users seeking treatment	0
long term	Users seeking treatment	0
Detox - outpatient	Users seeking treatment	0
MH Professionals	Users seeking treatment	0

Opiate Therapy	Opioid users seeking treatment	0
Rel.advisors	Users seeking treatment that participate in religious communities	0
12-step groups	Users seeking treatment	0
Peer support groups	Users seeking treatment	0
transportation	Users seeking treatment that have expressed need for transportation	0
employment support	Users seeking treatment that want to be part of the workforce	0
Educational support	Users seeking treatment with less than a high school education	0
Parenting education	Users seeking treatment who are parents	0
Housing Assistance	Homeless users seeking treatment	0
Insurance Access Assistance	Uninsured users	0

Total Population	Usage rates	Total Estimated # of users in community	Maximum Estimate of users seeking treatment	Total Estimated # of substance dependent or abusers in community
0				
Alcohol	22.9%	0	0	0
Cannabis	15.9%	0	0	0
Cocaine	1.6%	0	0	0
Methamphetamine/amphetamines	1.8%	0	0	0
Opiates	6.7%	0	0	0
Total number of Substance Users (%)		0	0	0

Combined Risk Factor	0.50	

Components	Maximum Community Need	Program Usage Rate	Adjusted community need	Observed Community Totals	Estimated Need	Units per Year
Promotion						
Social Marketing Advertisements	0	85%	0		0	Single advertisements
Media Advocacy Events	0	30%	0		0	Advocacy Events
Community Coalitions	0	100%	0		0	Coaltions
Prevention						
**Universal**						
School-based prevention programs	0	93%	0		0	1-hr long programs
Community-based prevention programs	0	12%	0		0	1-hr long programs
Faith-based prevention programs	0	9%	0		0	Short term programs
Workplace prevention programs	0	2%	0		0	Short term programs
**Selective**						
Mobile outreach services	0	58%	0		0	Texts &/or Alerts
Housing Voucher programs	0	42%	0		0	Individual vouchers
**Indicated**						
Needle Exchange	0	60%	0		0	Needle Exchange Locations
Prescription Drug Disposal Events/Locations	0	60%	0		0	Drug Disposal Outlets
Referal						
Adult Drug Courts	0	50%	0		0	Drug Courts
Primary Care Doctors w/ SA training	0	10%	0		0	Doctors
Social Workers	0	87%	0		0	Social Workers
Youth Drug Court	0	50%	0		0	Drug Courts
MH Awareness Trained Police	0	25%	0		0	Police Officers
Employer/EAP	0	2%	0		0	Programs
Treatment						
**Inpatient**						
Detoxification	0	1%	0		0	Detox Locations
24-hour/Intensive Day treatment	0	2%	0		0	Treatment programs
Short-term (30 days or fewer)	0	2%	0		0	Treatment programs
Long-term (more than 30 days)	0	6%	0		0	Treatment programs
**Outpatient**						
Detoxification	0	1%	0		0	Locations
Counselors, Psychiatrist or Psychotherapist	0	74%	0		0	Individual professionals
Office based opiate substitution	0	25%	0		0	Locations
Recovery Support						
Religious or spiritual advisors	0	1%	0		0	Individual professionals
12-step groups	0	30%	0		0	Groups
Peer support groups	0	9%	0		0	Groups
Transportation	0	14%	0		0	Bus trips
Employment support	0	5%	0		0	Individual professionals
Educational support	0	14%	0		0	Classes
Parenting education	0	7%	0		0	Classes
Housing Assistance	0	7%	0		0	Individual professionals
Insurance Assistance	0	90%	0		0	Individual professionals
